# Exploring role of sleep aids in sleep problems in preschool children

**DOI:** 10.1038/s41598-023-33758-z

**Published:** 2023-04-24

**Authors:** Masanori Yamaguchi, Mikako Ishibashi, Yusuke Moriguchi, Hisashi Mitsuishi, Shoji Itakura

**Affiliations:** 1 Center for Baby Science, Doshisha University, Kizugawadai, Kizugawa-shi, Kyoto, 619-0225 Japan; 2 Department of Psychology and Humanities, Edogawa University, Chiba, Japan; 3 Graduate School of Letters, Kyoto University, Kyoto, Japan; 4 Department of Health and Sports Science, Kyoto University of Advanced Science, Kyoto, Japan

**Keywords:** Psychology, Human behaviour

## Abstract

It is well known that children use sleep aids, such as blankets or soft toys, at bedtime. However, there is a lack of understanding regarding the factors associated with their use and role in addressing sleep problems. This study investigated 96 Japanese children aged 40 to 47 months to examine these associations. We measured children’s stress (through a questionnaire and salivary cortisol [cortisol awakening response]), anxiety symptoms, behavioral problems, and temperament, and created a model to predict the status of sleep aid use. Furthermore, we explored the association between sleep aid use and children’s sleep problems as evaluated by their caregivers. We found that children who used sleep aids were more likely to experience anxiety symptoms. Moreover, most children used sleep aids even when they co-slept with their caregivers and/or siblings. Their use was not uniquely associated with sleep problems. These findings suggest that sleep aid serves as a defense against anxiety, including that caused by the absence of a caregiver, rather than as a substitute for a caregiver. Our study sheds light on their role and emphasizes the importance of viewing development within the complex interactive processes of humans and objects.

## Introduction

Sleep is a crucial factor influencing many aspects of child development, including health, behavior and cognition^1^. Consolidated nighttime sleep is specifically important for child development, and many researchers assume that social context, including parenting, is crucial for sleep development, specifically, the development of self-soothing ability^2–4^. For example, in some cultures, including the United States (US), children sleep in a separate room, and try to nurture their self-soothing ability to sleep alone^5,6^. However, it is overlooked that human development is also supported by inanimate objects^7^. For instance, it has long been recognized that children use inanimate objects (hereafter sleep aids), such as blankets, bundles of wool, and soft toys, when they go to bed^8,9^.

From around one to four years old, children gradually develop their stress coping skills, and this period can be characterized as the emergence of intrapersonal coping (i.e., voluntary coping actions) that supplements external and interpersonal coping (i.e., coping by caregivers and co-regulation with caregivers)^10^. The emergence of sleep aids overlaps with this age period; thus, it is possible that sleep aids may support this development. Moreover, children's sleep ranks high on the list of parental concerns^11^, and sleep aids are candidates for addressing these concerns^12^. From a theoretical and practical standpoint, it is important to examine the factors involved in sleep aid use and its effectiveness. Despite this, little is known about the characteristics of children who use sleep aids and their role in sleep.

Sleep aids are inanimate objects used during the wake/sleep transition^13,14^. Although each concept emphasizes a different aspect, the concept of sleep aid overlaps with transitional^8,15^, attachment^16,17^, and personified objects^18,19^, because they are likely to be used during the early stages of sleep. The objects observed in such studies were also similar. For example, in sleep aid and attachment object research, two main object types are known: clothes, such as blankets, and soft toys, such as stuffed animals^15,19,20^. Although few studies have investigated how they are used, clothes are mainly used by infants to stimulate the mouth and skin; while, soft toys tend to appear a little later and are used as social agents, such as children talking to them and giving them personality traits^15,19^.

Our first question concerns the characteristics of children who use sleep aids. There are two primary theories in this field; one is that sleep aids are used as a substitute for caregivers^21,22^ and the other is that sleep aids are used as a defense against anxiety^8,20,23^. Importantly, these are not mutually exclusive theories, because needing a family member’s assistance to fall asleep is associated with higher anxiety severity in preschool children^24,25^.

Data, which support these views have been accumulating. For example, sleep aids are more likely to be observed in cultures where sleeping alone is more common^26–28^. Similar findings have been reported, even within the same culture, regarding sleep aids being more likely to be observed in families where sleeping alone is predominant^14,22,29^. Wolf and Lozoff^30^ demonstrated that children who fell asleep alone were more likely to use sleep aids than those who fell asleep in a caregiver's presence. Thus, these results seem to be consistent with both theories of sleep aids acting as a defense mechanism against anxiety, and as well as a substitute for caregivers.

However, some studies suggest that sleep aids may not always be able to be a substitute for caregivers. For example, some children used sleep aids, even when their caregivers were present^31,32^. Preschoolers, from cultures where they shared a bed with their caregivers, such as South Korea, were more likely to use stuffed animals at bedtime than US preschoolers^28^. Furthermore, some children slept with their stuffed animals, while sleeping next to their caregivers in Japan^18^. These results suggest that the two theories could be separable, and sleep aids are not always a substitute for caregivers. This calls for research examining the factors associated with the use of sleep aids, such as individual characteristics.

Our second question pertains to whether the use of sleep aids is associated with children’s sleep problems. Many studies have examined only the association between sleep aids and sleep arrangements–sleeping alone or cosleeping^22,26–28^. However, few have examined how the use of sleep aids is associated with children’s sleep^33^. To the best of our knowledge, only seven studies^5,12,14,29,34–36^ have previously examined this association.

Three studies have reported a positive association between the use of sleep aids and infants’ sleep quality. Anders et al.^5^, who examined the development of US children’s sleep longitudinally during the first year of life, reported that infants who used sleep aids slept longer at the age of three months, and that children who were rated by their caregivers as having no sleep problems at eight months were more likely to use sleep aids. Paret^34^ interviewed 80 mothers of nine-month-old infants through telephone or in-person meetings, and found that children who used sleep aids woke up less frequently during the night and slept longer. Hayes et al.^29^ found that US children aged three to five years who had used sleep aids during infancy were less likely to seek their parents when falling asleep, feeling afraid of the dark, or waking up during the night. However, Fukumizu et al.^14^ showed that sleep aid use was not related to sleep-related nighttime crying among infants (three- to six-months old) and toddlers (18- to 21-months old). Moreover, Burnham et al.’s intervention study^35,36^ demonstrated that providing mothers’ shirts to infants did not have a consistent effect on their sleep.

Two studies examined this association in preschoolers. Kushnir and Sadeh^12^ successfully improved sleep problems of four to six-year-old Israeli children with severe nighttime fears after a four-week intervention using stuffed animals (i.e., Huggy-Puppy intervention). These results suggest that the use of sleep aids effectively reduces anxiety during sleep, resulting in better sleep quality. However, Fukumizu et al.^14^ reported that Japanese children aged 36 to 41 months with sleep-related nighttime crying tended to use sleep aids more. Although the authors discussed the association between immature emotion/anxiety regulation and sleep-related nighttime crying, they did not explicitly discuss the advantages and disadvantages of sleep aid use. Thus, there is limited data and a lack of clarity regarding the relationship between sleep aid use and sleep, especially in preschoolers.

Furthermore, most studies evaluated a simple effect of sleep aid use on sleep. Therefore, we considered that exploring moderating factors could be useful for understanding the association between sleep aid use and sleep among preschoolers. For example, anxiety symptoms should also be considered as a potential factor. As discussed earlier, sleep aid use may be associated with anxiety^8^; evidence has shown an association between anxiety symptoms and sleep problems^24,25,37,38^. Moreover, preschoolers who refused to sleep alone showed higher anxiety severity^24,25^. Some studies reported an association between children’s fragmented sleep and cosleeping^39^. Studies that reported an association between sleep aid use and consolidated sleep were conducted in cultures where the rate of bed-sharing was low (23% in the US and 6% in Israel^40^). A similar pattern was found in cultures where children share a bed or futon mattress with their caregivers (54% in Japan^40^). Fukumizu et al.^14^ reported that “almost all the toddlers and preschoolers coslept in or next to the adult bed” (p. 221). Even though they found that infants without sleep-related nighttime crying were significantly more likely to sleep in separate, dedicated child beds than were those experiencing nighttime crying^14^. Thus, controlling for potential factors, including co-sleeping and anxiety, may be useful to understand the unique relationship between sleep aid use and children’s sleep.

Also, how sleep aids are used may be important, specifically for preschool-aged children. Kushnir and Sadeh’s^12^ findings suggest that the personification of stuffed animals might be critical. According to studies on play therapy, personification helps children express their emotions^41^. Moreover, Winnicott and Stevenson^20^ stated that “there was a transfer of emotion onto the inanimate object, which produced immediate comfort” (p. 212). Although Fukumizu et al.^14^ also found that most children used stuffed animals or puppets; however, they did not examine whether the children personified their sleep aids. It is important to note that no study has directly examined the effect of personification on children’s sleep. Thus, it is worth examining the effect of personification of sleep aids on children’s sleep.

In this study, we investigated 96 preschool-aged Japanese children, an age group on which previous studies are scarce and have not yielded clear results. First, we explored individual characteristics associated with sleep aid use. In theory, sleep aids can be regarded as stress-coping strategies, but the associations between sleep aid use and stress or anxiety have rarely been empirically and directly tested. Therefore, we measured variety of variables, including stress, anxiety symptoms, behavioral problems, and temperament through questionnaire and salivary cortisol level. Cortisol is an end-product of the endocrine system, the hypothalamus–pituitary–adrenal (HPA) axis. It rapidly increases within 30 min after waking up in the morning, and then gradually decreases (cortisol awakening response)^42,43^, and reflects the activity of the HPA axis. Cortisol reactivity is reported to be associated with anxiety symptoms among adults and adolescents^44^. However, this association has not been elucidated for preschool-aged children. Thus, we explored the association of both cortisol and anxiety with sleep aid use. If children use sleep aids to cope with stress or anxiety as mentioned in the theories, sleep aid use would be associated with stress and anxiety. We also checked the association between sleep aid use and sleep arrangements. According to the theory that sleep aid served as a substitute for caregivers, sleep arrangements and/or the presence of caregivers until children fall asleep^30^ would be associated with sleep aid use. Second, to explore the association between sleep aid use and children’s sleep, we asked caregivers to rate their children’s sleep problems. By creating a model, we estimated their effect by controlling for other potential factors. Our focus was on examining whether sleep aid use was uniquely associated with sleep problems. Thus, we did not develop a specific hypothesis. Moreover, we interviewed children to determine whether they personified their sleep aids, and focused on the effect of the interaction between sleep aid use and personification. If personification of sleep aid is a critical factor for children’s sleep problems, we would find a significant effect of the interaction between sleep aid use and personification.

## Methods

### Participants

This study was conducted between August 2021 and July 2022. Children and their caregivers registered in the participant pool housed by the Doshisha University Center for Baby Science were recruited through telephone or e-mail. A total of 100 preschool-aged Japanese children between 40 and 47 months of age and their caregivers, who lived in Kansai, Japan visited our laboratory and were interviewed. At the end of the session, the caregivers were asked to fill in a questionnaire booklet and collect their children’s saliva at home. Four caregivers did not return the questionnaire. Thus, the final sample consisted of 96 child-caregiver pairs (Children: *M*_age_ = 44.90 months, *SD* = 1.95 months, *n*_girl_ = 48, Caregivers: *M*_age_ = 36.54 years, *SD* = 9.95 years, *n*_mother_ = 93). Our survey protocol was approved by the Ethics Committee of Doshisha University (protocol number 20007), and was performed in accordance with the Declaration of Helsinki. Informed consent has been obtained from the parents for the participation of their children.

### Materials

The preschool anxiety scale was used to assess children’s anxiety symptoms^45^. The caregivers rated the extent to which each statement in the questionnaire applied to their children on a five-point scale. Child behavior checklists (CBCL) were used to measure children’s problematic behaviors^46,47^. It had two subscales, internalized and externalized problem behaviors, which were rated by the caregivers on a three-point scale. The children’s behavior questionnaire short form (CBQ) was used to rate the parental view of the children’s temperament^48,49^ on a seven-point scale. Three major factors were calculated: negative affectivity, surgency, and effortful control. Children’s sleep problems were assessed using the Japanese sleep questionnaire for preschoolers (JSQ-P)^50^, developed for assessing sleep disturbance and problematic sleep habits among Japanese preschoolers. The scale consists of 39 items, and the caregivers were asked rated their children’s sleep problems on a six-point scale. The parenting stress index (PSI)^51^ was used to measure the caregivers’ parenting stress. The imaginary companion questionnaire^52^ was used to see whether children personified their sleep aids. After reading an example of a child who personified a stuffed animal, the caregivers judged whether their children personified sleep aids, and if applicable, indicated what the object was. Along with these questionnaires, we also asked about demographic information (e.g., family income and educational background), the frequency of caregivers’ presence until the child fell asleep every week (never, 1–2 days, 3–5 days, and almost daily), room-sharing partner, bed-sharing partner, and sleep aid use. For sleep aid use, we asked “How many days a week does your child take a stuffed animal, toy or object to bed to sleep?”^29^, and if applicable, “What kind of objects does your child take to bed?” A comment box was provided so that caregivers could freely describe the details regarding their children’s sleep aids. The possible score ranges for these questionnaires are shown in Table 1.

**Table 1:** Descriptive statistics for all variables according to sleep aid use: means followed by standard deviations.

	Did not use sleep aids		Used sleep aids in the past		Use sleep aids currently		Total		
	*M*	*SD*	*M*	*SD*	*M*	*SD*	*M*	*SD*	
Child's age [range = 42–47]	45.19	1.87	46.00	1.41	44.54	1.99	44.90	1.95	
Anxiety score [range = 0–112]	30.92	16.34	35.69	16.45	38.20	14.45	35.22	16.00	
Child Behavior Checklist score [range = 0–200]	22.19	18.87	27.29	16.75	25.25	20.16	24.23	19.02	
CBQ (Negative affectivity) [range = 1–7]	3.73	0.58	4.19	0.86	3.98	0.73	3.90	0.70	
CBQ (Extraversion/Surgency) [range = 1–7]	4.34	0.89	4.01	1.12	4.23	0.83	4.26	0.88	
CBQ (Effortful Control) [range = 1–7]	5.23	0.49	5.34	0.73	5.17	0.53	5.20	0.53	
Sleep problem score (JSQ-P) [range = 39–234]	70.25	15.08	69.26	17.04	74.33	15.36	72.39	15.48	
Cortisol (waking) [nmol/L] [range = 1–37]	6.21	8.68	5.59	6.44	6.00	7.22	6.04	9.81	
Cortisol (AUCg) [range = 55.50–1061.25]	342.07	346.71	285.24	317.66	331.11	284.58	331.94	406.76	
Cortisol (AUCi) [range = -633.75–828.75]	60.27	280.47	28.14	252.94	61.12	229.96	58.43	324.21	
Caregiver's age [range = 28–50]	36.96	5.61	36.62	5.25	37.09	5.15	37.01	5.95	
Caregiver's educational background [range = 1–4]	2.56	0.66	2.71	0.49	2.58	0.70	2.58	0.70	
Family income [range = 1–4]	2.38	0.63	2.57	0.53	2.37	0.62	2.39	0.65	
Parenting Stress Index score [range = 78–390]	165.66	37.70	170.29	34.69	174.96	38.38	171.05	38.04	
Caregiver’s presence until sleep onset [range = 1–4]	3.59	0.89	4.00	0.00	3.62	0.81	3.64	0.82	

	*n*	%	*n*	%	*N*	%	*n*	%	
Child's sex	Boy	16	16.67	2	2.08	29	30.21	47	48.96
	Girl	20	20.83	5	5.21	22	22.92	47	48.96
Personification	Never/Past	26	27.08	2	2.08	35	36.46	63	65.63
	Current	10	10.42	5	5.21	16	16.67	31	32.29

One girl’s and one boy’s caregiver did not report sleep aid use and hence, the total number of participants was 94 for the child’s sex and personification. Means and standard deviations were estimated using multiple imputations. The following variables were used as dummy codes: caregiver’s educational background (1 = high school, 2 = junior college, 3 = 4-year-college/university or graduate school), family income (1 = less than ¥3,000,000, 2 = ¥3,000,001—¥7,999,999, 3 = ¥8,000,000–¥19,999,999, 4 = more than ¥20,000,000), and caregiver’s presence (1 = never, 2 = 1–2 days/week, 3 = 3–5 days/week, 4 = almost daily). CBQ = Child Behavior Questionnaire. Range indicates possible score ranges for questionnaires and observed score ranges for the other variables. CBCL and sleep problem scores (JSQ-P) had cutoff points. Nine percent of children were in the clinical range of the CBCL, and 25% were in the clinical range of the JSQ-P, which were consistent with previous reports^46,50^.

We conducted a semi-structured imaginary companion interview in our laboratory. After sharing a story as an example of a child who personified objects with the children, we asked them whether they talked to their sleep aids and attributed some personality traits to them. If they currently did so and their reports were consistent with their caregivers’, we coded the child currently personified their sleep aids.

To measure children’s salivary cortisol levels, we used an IPRO oral fluid collector (OFC; IPRO Interactive, Oxfordshire, UK). Saliva samples were assessed for cortisol concentrations using an IPRO lateral flow device (IPRO Interactive, Oxfordshire, UK) point-of-care system, which has been previously validated using ELISA analysis^53,54^.

### Procedure

Recruiters asked them to bring their sleep aids (if not applicable, favorite toys) to the laboratory. Participants conducted an imaginary companion interview to examine whether the children personified their sleep aids. After completing the interview, the interviewer asked their caregivers to fill in the questionnaire booklet at home and instructed them on how and when to collect their children’s saliva. Caregivers collected children’s saliva three times daily (immediately after waking, 30 min later, and 45 min later) according to the instructions of the IPRO interactive. The interviewer asked them not to eat or drink anything except water for 45 min after waking^43^. We confirmed verbally whether they appropriately collected the samples when they delivered them (six caregivers declared that they failed to follow the timing of saliva sampling). The saliva was immediately processed, and cortisol levels were measured (Of the three samples, at least one sample was insufficient for analysis for 12 participants). Data that were not reliable (samples from those who did not follow the instructions and those that did not provide a sufficient amount of saliva for analysis) were replaced and treated as missing values. The cortisol awakening response is a reliable endocrine marker of the hypothalamus–pituitary–adrenal (HPA) axis, and was indexed by two areas under the curve (AUCi and AUCg)^55^. AUCi represents the sensitivity of the HPA axis system, and AUCg represents the total secretion of cortisol within the first 45 min of waking up^55^. Children’s salivary cortisol levels significantly increased 30 min after waking up (*M* ± *SE* = 7.86 ± 0.71 [nmol/L], *p* = 0.004), and decreased within 45 min back to the same level (5.83 ± 0.52, *p* = 0.896) as waking up (5.74 ± 0.57), which was consistent with previous reports^43,56^.

### Data analysis

As there are some missing values, which can be regarded as missing at random (at a maximum of 17.71%, see Fig. S2), we estimate the correlation coefficients and regression coefficients by multiple imputations. A total of 50 completed datasets are created using 30 iterations with the predictive mean matching technique and integrated using Rubin’s rule and the MICE package^57^.

Sleep aids are defined as inanimate objects that children bring to their bed or futon to help them sleep. Since we did not directly observe the child during sleep onset, we include almost everything the caregivers believed was necessary for the children to sleep. We excluded three children because their caregivers believed that their children did not use toys for sleep although their children brought toys to bed. Two children brought their unspecified toys to their beds, put them away after playing with them for a while, and then fell asleep. One child brought only newly purchased toys to bed for a few days, and after getting bored with them, he stopped bringing them. We code these reports as “never” having sleep aids. We categorize sleep aid use into three groups: never used sleep aids, used sleep aids in the past, and currently using sleep aids.

Before creating the models, we compute Spearman’s rho correlation matrix to reduce the number of variables, because most are not normally distributed. Based on the matrix (Fig. S1), we decide to use the total anxiety score, CBCL score, and sleep problem score because they are intercorrelated (Spearman’s *ρ* = 0.242 to 0.966), and the total scores represent each subscale. For the CBQ score, we used three subscales (negative affectivity, extraversion/surgery, and effortful control) because they should be conceptually distinguished and not strongly correlated. Hence, we reduce the number of variables to 18: children’s age in months and sex, caregiver’s age and educational background, family income, three CBQ subscale scores, the frequency of caregivers’ presence until children’s sleep onset (days per week), sleep aid use (never vs. past vs. currently), personification (never/past vs. currently), sleep problem score, anxiety score, CBCL score, PSI score, cortisol at waking, AUCi, and AUCg.

To explore the factors associated with sleep aid use, we create a multinomial logistic regression model using 15 variables (we did not use the personification variable) as predictors. To explore the factors associated with sleep problems, their scores are modeled by all variables along with the interaction term between sleep aid use and personification, assuming a Poisson distribution.

Model selection is conducted based on the Akaike and Bayesian information criterion (AIC and BIC). For this, we explore the minimum AIC model for each completed dataset (*n* = 50) and count the number of times each variable survived in the minimum AIC model (Table S1). Then, we create several models using the variables that frequently survive in the minimum AIC models, compute the mean AICs and BICs, and decide on the final models. To predict the sleep aid use, we select the minimum AIC model (*M* = 172.1) that include child’s age and anxiety symptom because the minimum BIC model (*M* = 184.0) include only the age and was not a significant predictor. To predict sleep problem, we select the minimum BIC model (*M* = 775.9) that include the frequency of caregivers’ presence until children’s sleep onset, anxiety symptoms, and CBQ subscales (negative affectivity, extraversion/surgency, and effortful control).

The datasets and R codes analysed during the current study are available in the Open Science Framework (OSF) repository (https://osf.io/n5d3y/?view_only=b80dc5bac9cb4dec9787f662aee476e2).

### Ethical statements

Our survey protocol was approved by the Ethics committee of Doshisha University, protocol number 20007, and was also performed in accordance with the declaration of Helsinki. Informed consent have been obtained from the parents for the participation of their children.

## Results

### Cosleeping and sleep aids

A total of 51 (54.26%) children currently used sleep aids and 7 (7.45%) used them previously, while the remaining (38.30%) never used them. The children who had used them in the past stopped using them more than four months before the study was conducted. Most sleep aids were stuffed animals (88.24%), and the rest were clothes, dolls, and other toys. All the children slept with their caregivers in the same room. There were 21 (22.11%) children who slept in their bed or a futon mattress, and the others slept with their caregivers in the same bed or futon. Moreover, 77 (80.21%) caregivers reported that they were present until their children’s sleep onset, almost daily. Neither bed-sharing (Fisher’s exact test *p* = 0.441, Cramer's *V* = 0.141) nor the frequency of caregiver’s presence until the child’s sleep onset per week was associated with sleep aid use (Fisher’s exact test *p* = 0.776, Cramer's *V* = 0.156).

### Predictors of sleep aid use

The minimum AIC model (Table 2) showed that children who are currently using sleep aids had higher anxiety symptom scores than those who had never them. Children’s age was not a significant predictor. See Table 1 for the descriptive statistics for all variables.

**Table 2:** Results of the minimum AIC Poisson regression model for sleep aid use.

	*B*	*SE*	*z*	*df*	*P*	95%CI	
						Lower	Upper
In the past							
(Intercept)	− 1.83	0.51	− 3.58	87.17	.001	− 2.83	− 0.83
Child’s age	0.54	0.55	0.99	86.61	.323	− 0.53	1.61
Anxiety symptoms	0.33	0.46	0.71	78.09	.482	− 0.58	1.24
Currently							
(Intercept)	0.36	0.23	1.57	83.70	.120	− 0.09	0.82
Child’s age	− 0.35	0.23	− 1.50	84.99	.138	− 0.81	0.11
Anxiety symptoms	0.55	0.27	2.04	67.19	.045	0.02	1.08

The reference was “did not use sleep aids.” The model was estimated using multiple imputations (see "Methods" section).

### Predictors of sleep problems

**Figure 1:**
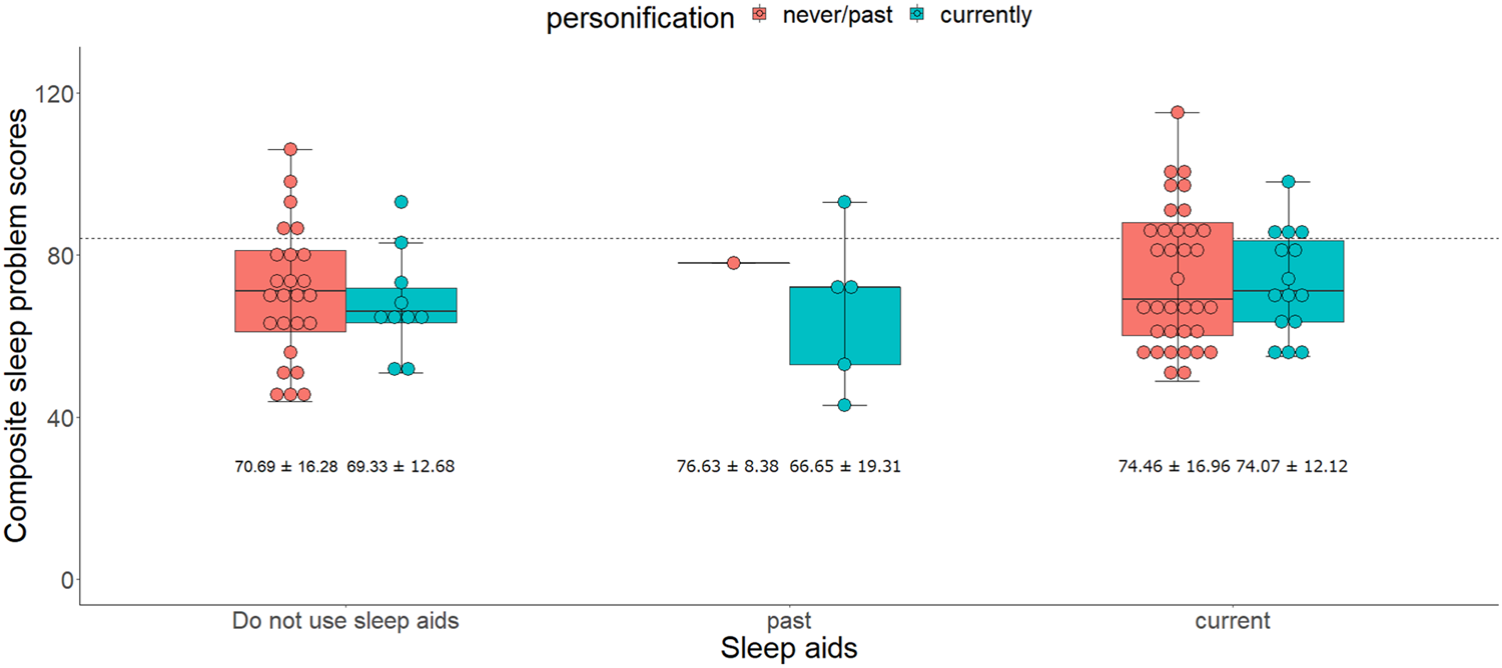
Interaction effect between sleep aid use and personification on sleep problems. The interaction effect term did not significantly predict sleep problem scores. The horizontal line indicates the cut-off value (84) of the Japanese sleep questionnaire for preschoolers. The numbers below the boxes indicate mean ± standard deviation.

The minimum BIC model showed that the presence of frequent caregivers until sleep onset, greater anxiety symptoms, greater negative affectivity, and extraversion/surgency were associated with more sleep problems. Effortful control was associated with fewer sleep problems (Table 3). Although we exploratorily included the interaction effect between sleep aid use and personification in the minimum BIC model, they were not significant predictors (Table S2 and Fig. 1).

**Table 3:** Results of the minimum BIC Poisson regression model for sleep problems.

	*B*	*SE*	*z*	*df*	*P*	95%CI	
						Lower	Upper
(Intercept)	4.27	0.01	346.17	84.73	< .001	4.25	4.30
Caregiver’s presence until sleep onset	0.03	0.01	2.66	85.96	.009	0.01	0.06
CBQ (Negativity)	0.05	0.01	3.20	70.96	.002	0.02	0.07
CBQ (Surgency)	0.08	0.02	4.76	70.02	< .001	0.04	0.11
CBQ (Effortful)	− 0.07	0.01	− 5.08	79.84	< .001	− 0.09	− 0.04
Anxiety symptoms	0.09	0.02	5.01	57.15	< .001	0.06	0.13

The model was estimated using multiple imputation (see "Methods" section).

## Discussion

The first aim of this study was to explore the factors associated with sleep aid use. According to Winnicott^8^, sleep aids can function as a defense against anxiety, and other researchers have suggested that they can function as a substitute for their caregivers^21,22^. However, 39 (41.94%) children were currently using sleep aids, even though they co-slept with their caregivers and/or siblings in the same bed or futon. Moreover, 40 (42.55%) children were currently using sleep aids, although their caregivers were present until they fell asleep. These results suggest that sleep aid is not always a substitute for caregivers, because the children used sleep aids, while their caregivers were present.

We found that anxiety symptoms were significantly associated with sleep aid use. We interpreted this finding to indicate that children who easily become anxious might also experience anxiety at bedtime and use sleep aids to manage it. We suggest that sleep aids might have a special, possibly anxiety-related function apart from providing comfort. Another possibility was that children used sleep aid to cope with separation anxiety. Although most of the caregivers in the current study co-slept with their children, they may have temporarily left the bed to complete household chores after putting the child to bed. If so, the children could have experienced separation anxiety when they woke up in the middle of the night. This could not be tested in this study and future studies are needed to investigate this possibility. Considering that previous studies reported an association between sleep aid use and sleeping alone^5,22,26–30^ and the results of the current study, it may be better to consider sleep aid as a coping strategy against anxiety, including that caused by the absence of a caregiver^24,25^, rather than a substitute for a caregiver.

Our second aim was to explore the effects of sleep aid use on children’s sleep problems. Consistent with the findings of Fukumizu et al.^14^, who found an association between sleep-related nighttime crying and sleep aid use, we also found that children who were currently using sleep aids were rated by their caregivers as having more sleep problems, compared to those who never used sleep aids (see Table 1). However, we did not find a unique association between sleep aid use and sleep problems when controlling for anxiety scores or temperament. Therefore, we interpreted that sleep problems may be associated with anxiety symptoms^14,24,25,37,38^ or other factors, and that sleep aid use itself did not cause sleep problems. Rather, children may use sleep aids to cope with their anxiety and/or sleep problems caused by it.

To explore moderator variables, we analyzed the effect of personification. However, we could not find a significant effect resulting from the interaction between sleep aid use and personification of sleep problems; although, Kushnir and Sadeh^12^ and others^20,41^ suggested that the personification of objects is helpful for children to overcome or manage their anxiety or fear, including nighttime fear. We considered three possible reasons for this result. First, this may be due to the small sample size. Among the 51 children who were currently using sleep aids, only 16 personified them. As shown in Fig. 1, the direction was the same as Kushnir and Sadeh’s^12^, and found that among children who were currently using sleep aids, those who personified these objects were rated as having fewer sleep problems. Second, there may be other important factors that we did not assess. For example, Kushnir and Sadeh^12^ noted the importance to assess “the role of non-therapeutic factors such as involvement in the assessment and discussion of the problem, positive expectations, providing gift to the child (the doll)” (p. 74). Moreover, Thomas et al.^11^ mentioned “Although sleep aids may be successfully used in conjunction with an empirically supported behavioral intervention, the independent presence of a sleep aid is not likely to foster the development of self-soothing skills” (p. 255). Finally, the function of sleep aids differs according to sleep arrangements. Previous studies that showed the sleep aids’ effectiveness were conducted in countries where sleeping alone is common^5,29,34^. Kushnir and Sadeh’s^12^ study was conducted in Israel, where it was predominant^40^. Thus, children in these countries may actively use sleep aids to comfort themselves. Whereas, children in countries such as Japan, may not have enough experience to comfort themselves using sleep aids, because their caregivers may actively comfort them. Fifty-five percent of caregivers considered co-sleeping should be stopped by the time their children enter elementary school, and 38% believed it should be stopped when their children enter junior high school^58^. Children in such countries may develop emotional regulation skills which is associated with nighttime fear^59^, and are able to cope with anxiety and fear on their own, before they stop sleeping with their caregivers. If so, they may stop using sleep aids before realizing their benefits. In conclusion, although we could not find evidence that the personification of sleep aids helps reduce sleep problems in children, Burnham et al.^35,36^ reported that providing infants their mothers' shirts had no consistent effect on them, suggesting that tactile and odor features cannot calm children and foster self-soothing abilities^11^. Therefore, it would be worthwhile to examine the effects of personification on sleep in future studies.

Notably, this study had several limitations. First, although children’s sleep problems were rated by their caregivers, caregivers’ subjective reports on their children’s sleep might be slightly different from objective measurements, such as actigraphy^60^. Second, most current findings were from our exploratory analysis; therefore, replicability and generalizability are not well established. Third, because of the nature of correlational studies, the longitudinal correlation between sleep aids and problems remains unclear. Further studies are required to more rigorously examine whether sleep aid is uniquely associated with sleep, as well as to explore moderator variables. Although we did not collect details on how children use sleep aids (e.g., just placing it nearby and/or touching it), this may have an effect on children’s sleep quality. The relationship between sleep aid use and anxiety symptoms is also interesting; whether and how sleep aids can reduce children’s anxiety will be addressed in future work. Despite these limitations, our study sheds new light on the role of sleep aids in preschool-aged children and highlights the need for research. That is, although previous studies have accumulated evidence that social factors or contexts, including parenting, are associated with children’s development of sleep^2,3^, our findings suggest that sleep aids might play a different and unique role apart from being substitutes for caregivers at bedtime. This reemphasizes the importance of viewing human development, specifically, sleep, within the human–human and human-object contexts^7,36^. Moreover, our findings suggest a relationship between sleep aids and sleep problems. We hope that studies on sleep aid will be conducted in the future to take into account cultural differences and moderator variables.

## Supplementary Information

Supplementary Information.

## Data Availability

The datasets and R codes analysed during the current study are available on the Open Science Framework (OSF) repository, (https://osf.io/n5d3y/?view_only=b80dc5bac9cb4dec9787f662aee476e2).
