# Supplementary material for: Exploring role of sleep aids in sleep problems in preschool children
Source: Sci Rep. 2023 Apr 24;13:6612. doi: 10.1038/s41598-023-33758-z (PMC10125968; doi:10.1038/s41598-023-33758-z)
Supplement: Supplementary file 1 — Supplementary Information. [file 41598_2023_33758_MOESM1_ESM.docx]

**Figure S1.** Correlation matrix heatmap.

*
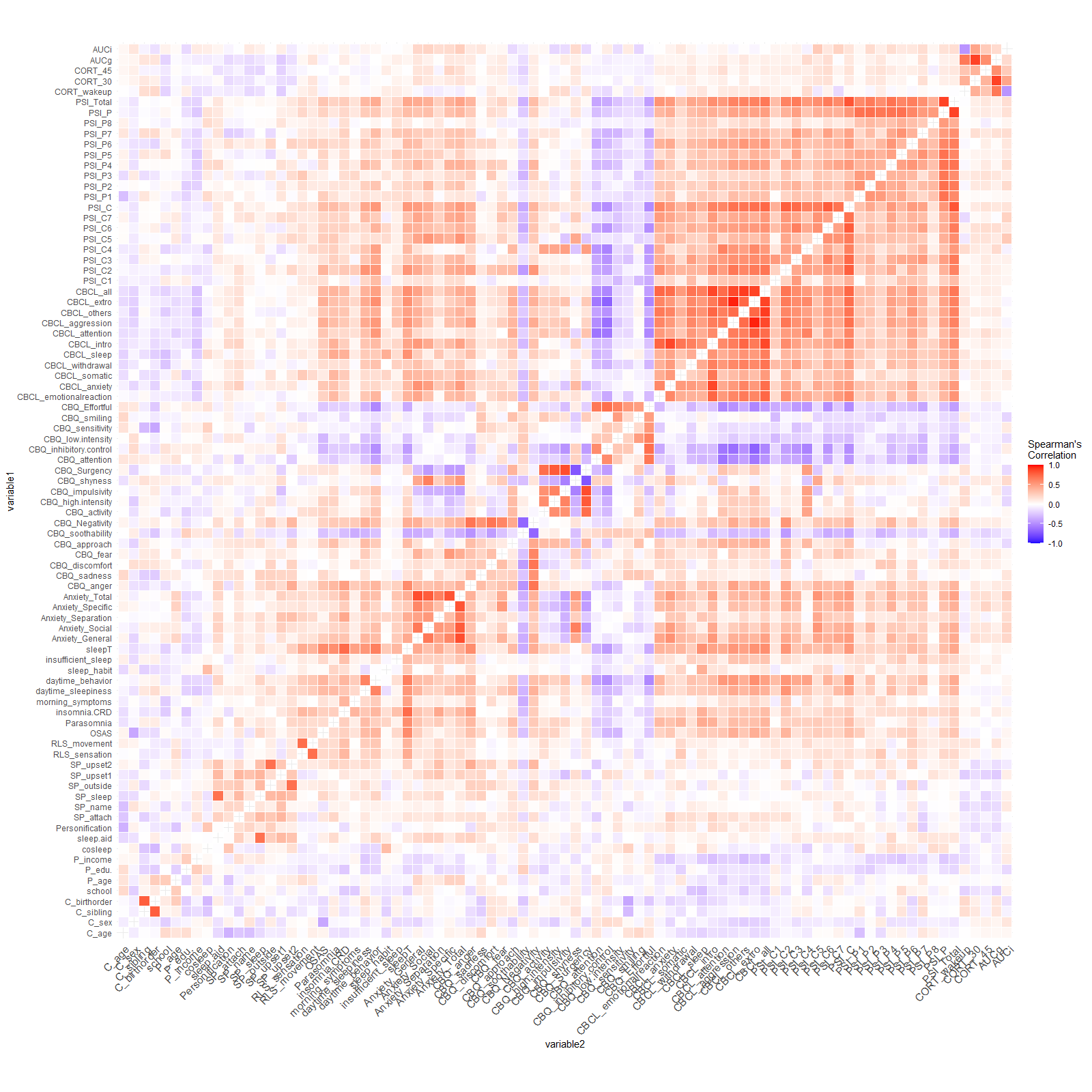
Note*. This figure shows that the subscales of each questionnaire were intercorrelated and that the total scores reflected the scoring trends of these subscales, except for the Child Behavior Questionnaire (CBQ). Readers can find all Spearman’s rank correlation coefficients (rho) and *p*-values using our datasets and codes.

**Figure S2.** Patterns of missing values.

*
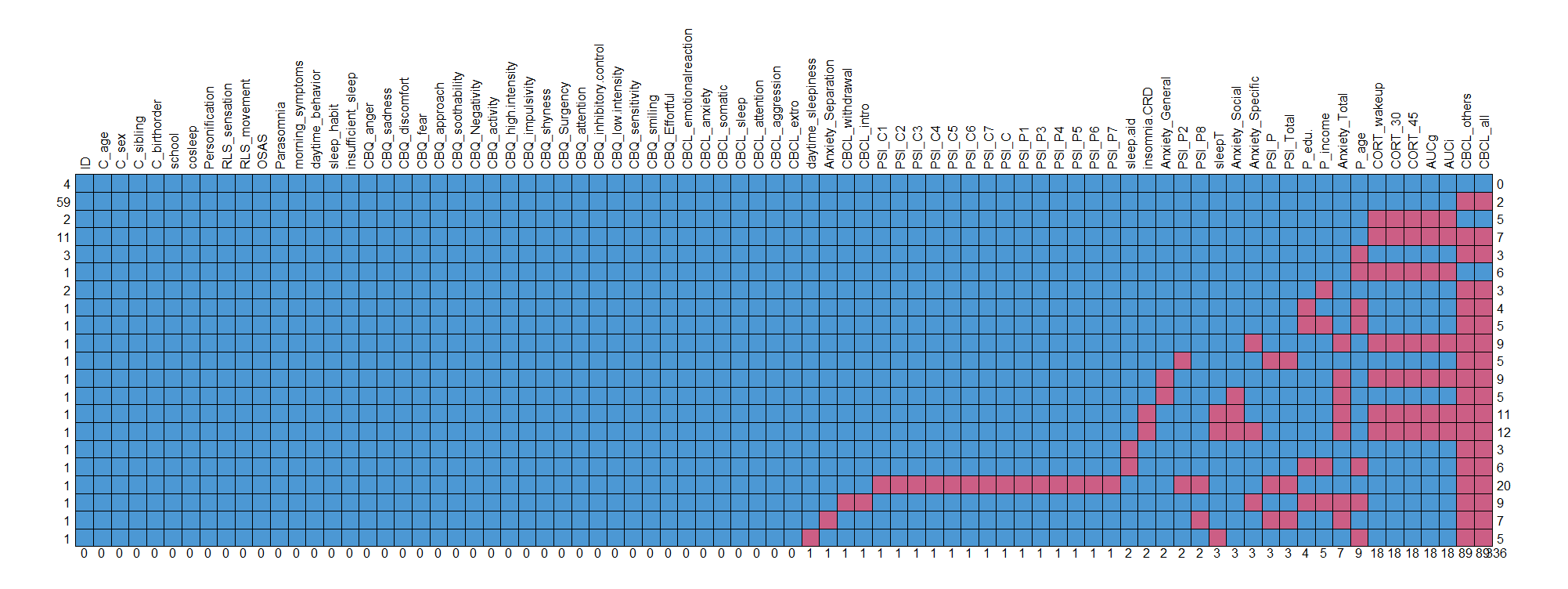
Note*. Red indicates the missing values.

**Table S1.** Model selection.

| **Predictors for sleep aid use** | Num of Survivals | AIC_mean_ | BIC_mean_ |
| --- | --- | --- | --- |
| Child’s age in month | 50 | 173.8 | **184.0** |
| + Anxiety symptoms | 41 | **172.1** | 187.4 |
| + Child sex | 24 | 172.9 | 193.4 |
| + Cortisol (AUCi) | 20 | 174.7 | 200.3 |
| + Cortisol (waking) | 18 | 176.7 | 207.5 |
| + Cortisol (AUCg) | 17 | 177.1 | 213.0 |
| + CBQ (Surgency) | 16 | 180.0 | 221.1 |
| + CBQ (Negativity) | 15 | 181.3 | 227.5 |
| + CBCL | 15 | 180.7 | 232.0 |
| + Caregiver’s presence until sleep onset | 6 | 182.4 | 238.8 |
| **Predictors for Sleep problems** | Num of Survivals | AIC_mean_ | BIC_mean_ |
| Caregiver’s presence until sleep onset | 50 | 896.7 | 901.8 |
| + CBQ (Negativity) | 50 | 849.2 | 856.9 |
| + CBQ (Surgency) | 50 | 835.1 | 845.3 |
| + CBQ (Effortful) | 50 | 794.4 | 807.2 |
| + Anxiety symptoms | 50 | 760.6 | **775.9** |
| + Personification | 46 | **760.4** | 778.4 |
| + Cortisol (AUCi) | 37 | 757.5 | 778.0 |
| + Child’s age in month | 28 | 756.9 | 780.0 |
| + Cortisol (waking) | 27 | 758.4 | 784.1 |
| + Cortisol (AUCg) | 25 | 757.9 | 786.1 |

*Note*. This table shows the stepwise model-selection process. The num of survival indicates the number of times that each variable survived in the minimum AIC models out of the 50 completed datasets. The minimum AIC/BIC models are indicated in bold.

**Table S2**. Results of the exploratory Poisson regression models that included personification and sleep aid use.

|  |  |  |  |  |  | 95%CI | |
| --- | --- | --- | --- | --- | --- | --- | --- |
|  | *B* | *SE* | *z* | *df* | *p* | lower | upper |
| (Intercept) | 4.29 | 0.02 | 173.35 | 74.63 | < .001 | 4.24 | 4.33 |
| Caregiver's presence until sleep onset | 0.04 | 0.01 | 2.85 | 80.81 | .006 | 0.01 | 0.06 |
| CBQ (Negativity) | 0.05 | 0.01 | 3.27 | 67.56 | .002 | 0.02 | 0.08 |
| CBQ (Surgency) | 0.08 | 0.02 | 4.88 | 66.78 | < .001 | 0.05 | 0.11 |
| CBQ (Effortful) | -0.06 | 0.01 | -4.55 | 74.39 | < .001 | -0.09 | -0.04 |
| Anxiety symptoms | 0.09 | 0.02 | 4.97 | 53.33 | < .001 | 0.06 | 0.13 |
| Personification | 0.03 | 0.11 | 0.32 | 46.56 | .751 | -0.18 | 0.25 |
| Sleep aid (past) | 0.00 | 0.03 | -0.11 | 72.22 | .916 | -0.07 | 0.06 |
| Sleep aid (current) | -0.03 | 0.05 | -0.58 | 76.81 | .561 | -0.11 | 0.06 |
| Sleep aid (past) × Personification | -0.11 | 0.13 | -0.85 | 57.17 | .400 | -0.36 | 0.14 |
| Sleep aid (current) × Personification | 0.00 | 0.06 | -0.03 | 76.84 | .974 | -0.12 | 0.11 |

*Note*. The model was estimated using multiple imputations (see Methods section).
